# Connecting within-host dynamics to the rate of viral molecular evolution

**DOI:** 10.1093/ve/vev013

**Published:** 2015-10-02

**Authors:** Kayla M. Peck, Carmen H. S. Chan, Mark M. Tanaka

**Affiliations:** ^1^Department of Biology, University of North Carolina - Chapel Hill,; ^2^School of Biotechnology and Biomolecular Sciences, University of New South Wales, Sydney, NSW, Australia and; ^3^Evolution & Ecology Research Centre, University of New South Wales, Sydney, NSW, Australia

**Keywords:** eevolutionary rate, virus evolution, within-host dynamics, mutation rate, influenza virus

## Abstract

Viruses evolve rapidly, providing a unique system for understanding the processes that influence rates of molecular evolution. Neutral theory posits that the evolutionary rate increases linearly with the mutation rate. The occurrence of deleterious mutations causes this relationship to break down at high mutation rates. Previous studies have identified this as an important phenomenon, particularly for RNA viruses which can mutate at rates near the extinction threshold. We propose that in addition to mutation dynamics, viral within-host dynamics can also affect the between-host evolutionary rate. We present an analytical model that predicts the neutral evolution rate for viruses as a function of both within-host parameters and deleterious mutations. To examine the effect of more detailed aspects of the virus life cycle, we also present a computational model that simulates acute virus evolution using target cell-limited dynamics. Using influenza A virus as a case study, we find that our simulation model can predict empirical rates of evolution better than a model lacking within-host details. The analytical model does not perform as well as the simulation model but shows how the within-host basic reproductive number influences evolutionary rates. These findings lend support to the idea that the mutation rate alone is not sufficient to predict the evolutionary rate in viruses, instead calling for improved models that account for viral within-host dynamics.

## 1 Introduction

The rate of molecular evolution is a fundamental facet of evolutionary processes. Although many complex factors can influence the evolutionary rate, the neutral theory of molecular evolution suggests that most fixations are, in fact, selectively neutral. Under this assumption, the mutation rate is the sole predictor of the evolutionary rate (Kimura 1984). While this theory has often been successful in estimating rates of evolution (Li, Tanimura, and Sharp 1987; Kimura 1991; Bromham and Penny 2003), its application to viruses is not straightforward. Some studies have found that many rapidly evolving viruses follow typical molecular clock dynamics (Gojobori, Moriyama, and Kimura 1990; Leitner and Albert 1999), whereas others have shown that neutral theory does not hold the majority of the time (Jenkins et al. 2002). The lack of consensus on whether viruses conform to the predictions of the neutral theory is complicated by the fact that viruses often have high mutation rates and complex life cycles.

Viruses have a wide range of mutation rates, with DNA viruses having rates as low as $10^{-8}$ substitutions per nucleotide site per cell infection (s/n/c) and RNA viruses having rates as high as $10^{-3}$ (s/n/c) (Drake 1993; Sanjuán et al. 2010). The high mutation rates of RNA viruses are matched by high evolutionary rates (Jenkins et al. 2002; Hanada, Suzuki, and Gojobori 2004); however, high mutation rates are expected to come with an elevated risk of extinction due to deleterious mutations arising more rapidly than can be removed by selection (Holmes 2003; Bull, Sanjuan, and Wilke 2007). This suggests that the evolutionary rate for viruses cannot increase indefinitely with increasing mutation rate but instead is limited by an upper bound. Indeed, it has been shown that while the neutral theory applies well to slowly mutating viruses (DNA and double-stranded RNA viruses), the linear relationship between mutation rate and evolutionary rate breaks down for faster mutating viruses (single-stranded RNA and retroviruses) (Sanjuán 2012). This discrepancy can be viewed as evidence in favor of viruses being close to an error or extinction threshold which restricts the evolutionary rate in the face of abundant transient deleterious mutations present in the population (Eigen 1993; Bull, Sanjuan, and Wilke 2005; Pybus et al. 2007). Sanjuán (2012) showed that the deviation from the predicted linear relationship can be explained by the effect of background selection, where neutral diversity is removed due to linkage with deleterious effects (Charlesworth, Morgan, and Charlesworth 1993; Orr 2000).

Sanjuán’s (2012) analysis revealed that deleterious mutations are important; however, the underlying model is general and therefore does not take into account differences in the within-host dynamics of individual viruses. As a result, while this model is a useful starting point, it produces the same prediction for viruses that may have similar mutation rates but drastically different life cycle strategies. For example, avian hepatitis B virus and human immunodeficiency virus (HIV) have similar mutation rates (2.0 $\times 10^{-5}$ and 2.4 $\times 10^{-5}$ (s/n/c), respectively (Sanjuán et al. 2010)) yet have evolutionary rates that are quite different (7.32 $\times 10^{-4}$ and 2.74 $\times 10^{-3}$, subsitutions/site/year (s/n/y), respectively (Sanjuán 2012)). These differences in the evolutionary rates may be due to differences in their within-host dynamics.

Viruses have evolved different strategies for entering and growing within hosts; they are cleared in a variety of ways by immune responses, and they vary in how they exit a host to be transmitted to the next. Previous studies examining the relationship between the within-host and between-host processes have shown that specific within-host parameters may influence the evolution of the virus population at the within-host and/or between-host scale, such as viral genomic architecture, replication rate (Luciani and Alizon 2009), life cycle (Duffy, Shackelton, and Holmes 2008), cell tropism (Hicks and Duffy 2014), cellular immune processes (Lemey et al. 2007; Luciani and Alizon 2009; Fryer and McLean 2011), and within-host competition (Lythgoe, Pellis, and Fraser 2013), as well as between-host parameters such as epidemiological dynamics (Scholle et al. 2013) and ecological factors (Hanada, Suzuki, and Gojobori 2004; Streicker et al. 2012).

The within-host dynamics of viruses are becoming increasingly well understood, with many models accurately predicting virus population trajectories through parameters such as virus growth rate, virus clearance rate, cell infection rate, and cell death rate (Nowak et al. 1996; Perelson et al. 1996; Baccam et al. 2006). Characterizing these dynamics has been useful for examining the efficacy of drugs/treatments (Neumann et al. 1998; Perelson and Ribeiro 2008), understanding the evolution of drug resistance (Rong et al. 2007), the roles of innate and adaptive immunity during infection (Pawelek et al. 2012), and the evolution of mutation rates (Regoes, Hamblin, and Tanaka 2013). Such models have now matured to the point where they can be used to better understand viral evolution.

Here, we develop two models to investigate the effect of within-host dynamics on the rate of molecular evolution in acute viruses. Given the short duration of infection, the dynamics of viral mutants within a host are transitory, but we are interested in understanding how this process affects the evolutionary rate at the between-host level. We begin with a simple analytical model similar to the Luria–Delbrück process in which mutants appear stochastically in a growing population. We include a new critical parameter: the within-host reproductive number. Our analytical model is useful for understanding the virus evolutionary rate as a function of this single parameter which summarizes within-host processes. To further enhance our understanding of the role of acute virus life cycles, we also develop a computational model that simulates evolution in a population of viruses that are target cell limited (Perelson et al. 1996). We compare the performance of our model incorporating within-host population dynamics to the deleterious mutation model implemented by Sanjuán (2012) to explain the evolutionary rates of a range of viruses. We also consider in more detail the specific case of influenza A virus, for which parameters have been estimated from patient data (Baccam et al. 2006).

Viruses provide a unique opportunity to study evolutionary rates because we can directly witness and measure them within our lifetime. Additionally, understanding the factors that influence these rates of evolution can offer insight into both life-cycle strategies and the epidemiology of currently circulating virus strains. The analytical and computational models presented here enhance our understanding of the rate of molecular evolution in viruses by highlighting the importance of within-host dynamics for the overall evolutionary rate.

## 2 Models and methods

### 2.1 Simple within-host analytical model

We begin with a simple within-host growth process in which a virus population grows exponentially and selectively neutral mutations appear randomly. We assume that new mutants are either neutral or deleterious. Neutral mutants can be lost by chance (i.e., through genetic drift), but deleterious mutations are assumed to be removed from the population immediately due to selection. We assume that transmission to another host occurs at the peak of infection (Bell et al. 2006) at some time $t_{p}$. Mutants that appear after the peak are doomed to extinction with high probability, and this allows us to neglect the post-peak dynamics as a first approximation. We note that this assumption would not be suitable for viruses such as HIV and hepatitis C virus (HCV), which cause chronic infections. We seek the proportion $p_{m}$ of the virus population that is made up of neutral mutants at this peak. We use this proportion to compute the rate of neutral substitution.

The growth of the virus population depends on the within-host reproductive number ($R_{0}^{\text{wh}}$) which is defined as the average number of second-generation infections produced by a single infected cell (Baccam et al. 2006). This is not to be confused with the between-host reproductive number $R_{0}$ (Heffernan, Smith, and Wahl 2005). We scale time such that 1 unit equals the generation time of an infected cell. In one cellular generation, the virus generates ρ new infected cells, but a fraction 1−λ of these are lost due to background selection, that is, because of the deleterious mutations carried by the new viruses. The reproductive number of the virus is therefore $R_{0}^{\text{wh}}=\rho \lambda$ where λ is the probability that a genome does not carry a deleterious mutation, given by

(1) $$\lambda =e^{-(1-\alpha )\mu G/s_{\text{H}}}$$

where α is the proportion of mutations that are neutral, μ is the per-site per-cell generation mutation rate, G is the genome size, and $s_{\text{H}}$ is the harmonic mean of deleterious effects (Orr 2000). Thus, the viral population grows according to $I_{0}e^{(\rho \lambda -1)t}$ where t is time measured in infected cell generations and $I_{0}$ is the initial number of infected cells.

Neutral mutants appear at a rate of αμ per site per cell per generation. If the dynamics of mutant viruses follow a stochastic linear birth–death process, the probability of ultimately surviving extinction (see Iwasa, Michor, and Nowak 2004) is

$$\{\begin{array}{ll} 1-\frac{1}{\rho \lambda } & \;\text{if}\;\;\rho \lambda >1\\ 0\; & \;\text{otherwise}\;. \end{array}$$

Using the same reasoning as in Luria and Delbrück (1943), the expected number of mutants at the peak of infection at time $t_{p}$ is

$$\alpha \mu (1-1/(\rho \lambda ))t_{p}I_{0}e^{(\rho \lambda -1)t_{p}}$$

assuming ρλ>1. Note that unlike the Luria-Delbrück model, we allow the average offspring number to take any positive value rather than restricting it to be exactly 2 (that is, $R_{0}^{\text{wh}}$ need not be 2), and we include the possibility that mutants are lost by stochastic extinction. Therefore, the proportion $p_{m}$ of mutants at the peak of infection at time $t_{p}$ is given by

(2) $$p_{m}=\;\{\begin{array}{ll} \alpha \mu \left(1-\frac{1}{\rho \lambda }\right)t_{p} & \;\text{if}\;\rho \lambda >1\\ 0 & \;\text{otherwise}.\; \end{array}$$

The duration of infection measured in years is $t_{p}/g$ where g is the number of cell generations per year.

The substitution rate $K_{\text{wh}}$ per year as predicted by our within-host analytical model is therefore

(3) $$K_{\text{wh}}=\{\begin{array}{ll} \alpha \mu \left(1-\frac{1}{\rho \lambda }\right)g & \;\text{if}\;\rho \lambda >1\\ 0 & \;\text{otherwise}\; \end{array}\;$$

where g is the average number of cell generations per year. Thus, our model accounts for within-host dynamics (through ρ) and deleterious mutation (through λ). This can be compared with the deleterious mutation model implemented by Sanjuán (2012) (see also Orr 2000):

(4) $$K_{\text{del}}=\alpha \mu \lambda g.$$

Note that the deleterious mutation model indirectly accounts for $R_{0}^{\text{wh}}$ through the cell generation time parameter g; a longer generation time increases the chance of producing more viruses and infecting more cells. However, our model is more explicit about the role of $R_{0}^{\text{wh}}$ (through the parameter ρ) in determining the evolutionary rate.

### 2.2 Within-host computational model

We develop a semi-stochastic computational model of an acute viral infection to complement the within-host analytical model. We begin with ordinary differential equations for a model in which the number of target cells is limited. The model structure follows that of Baccam et al. (2006) by defining the immune response implicitly in the infected cell death and virus clearance rates and forgoing the incorporation of a specific immune response. In these dynamics, the virus population declines when the target cell population is depleted. This is realistic because some immune responses peak along with, or are not detected until after, peak viral replication (Richman et al. 1976; Ennis et al. 1981; Baccam et al. 2006). By avoiding the complex and poorly understood immune system dynamics, target cell-limited models represent a simple way to accurately model virus infections and have been in wide use for exploring the kinetics of other viruses such as HIV, HCV, and HBV (Nowak et al. 1996; Perelson et al. 1996; Neumann et al. 1998). However, sophisticated models of virus evolution have been successful in exploring within-host parameters in the context of the immune response (Luciani and Alizon 2009).

#### 2.2.1 Infection within a single host

The dynamics are specified by a discrete-time stochastic version of the Baccam et al. (2006) model, expanded to include a neutral mutation process. Let T(t) be the number of target cells, I(t) be the number of cells infected by a wildtype virus, M(t) be the number of cells infected by a mutant virus, V(t) be the wildtype virus population, and W(t) be the mutant virus population, all at time t. Viruses infect cells with rate parameter β and are cleared at rate c per virion per unit time. Infected cells are cleared at rate δ per cell per unit time and viruses are produced at rate p per cell per unit time. The dynamics progress according to

(5) $$\begin{array}{l} T(t+1)=-B_{\text{wt}}-B_{\text{mut}}\\ I(t+1)=B_{\text{wt}}-D_{\text{wt}}\\ M(t+1)=B_{\text{mut}}-D_{\text{mut}}\\ V(t+1)=P_{\text{wt}}-C_{\text{wt}}\\ W(t+1)=P_{\text{mut}}-C_{\text{mut}}+A \end{array}$$

where the terms on the right hand sides are random variables described below. The subscripts wt and mut indicate terms involving wildtype and mutant viruses, respectively. The random variables give the numbers of new target cells infected by wildtype ($B_{\text{wt}}$) and mutant ($B_{\text{mut}}$) viruses; the numbers of cell deaths among cells infected by wildtype ($D_{\text{wt}}$) and mutant ($D_{\text{mut}}$) viruses; the numbers of viruses produced by cells infected with wildtype ($P_{\text{wt}}$) and mutant ($P_{\text{mut}}$) viruses; the numbers of wildtype ($C_{\text{wt}}$) and mutant ($C_{\text{mut}}$) viruses cleared by the immune system; and the number of new mutants that arise by mutation (A). Each term above is modeled stochastically using within-host parameters, with

(6) $$\begin{array}{l} B_{\text{wt}}∼\text{Poisson}(\beta \lambda TV),\;B_{\text{mut}}∼\text{Poisson}(\beta \lambda TW)\\ D_{\text{wt}}∼\text{Poisson}(\delta I),\;D_{\text{mut}}∼\text{Poisson}(\delta M)\\ P_{\text{wt}}∼\text{Poisson}(pI),\;P_{\text{mut}}∼\text{Poisson}(pM)\\ C_{\text{wt}}∼\text{Poisson}(cV),\;C_{\text{mut}}∼\text{Poisson}(cW)\\ A∼\text{Poisson}(\mu \alpha V). \end{array}$$

If any dynamic variables at the next time step become negative, they are reset to zero. All mutations are neutral except lethal mutations in the λ factor and the Poisson distribution permits more than one mutant to arise during the same time step.

**Figure 1.**
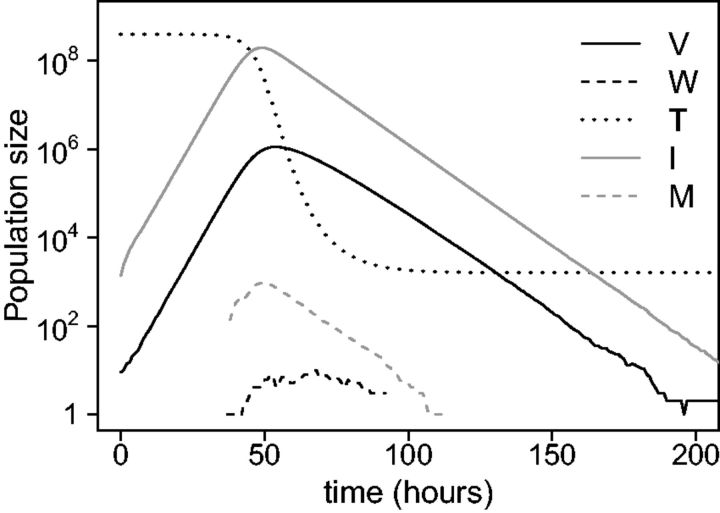
Population dynamics in a single infected host. Wildtype virus (V) and mutant virus (W) population within-host dynamics with corresponding target cell (T), wildtype virus-infected cell (I), and mutant virus-infected cell (M) populations shown for a single infection using default parameters (Table 1).

A summary of the parameters can be found in Table 1 along with default values used in the computational analyses. Each simulation is initialized with all variables set to zero except T which is set to $T_{0}=4\times 10^{8}$ and V which is set to $V_{\text{inoc}}=10$. Typical dynamics of the populations modeled in Equation (5) within a single infected host are shown in Fig. 1.

**Table 1.** Summary of infection parameters.

Parameter	Description	Default value (unit)	Source
β	Cell infection rate	$1.13\times 10^{-6}$ (${\text{h}}^{\text{-1}}$)	Baccam et al. (2006)
δ	Cell death rate	0.1 (${\text{h}}^{\text{-1}}$)^a^	Baccam et al. (2006)
p	Virus growth rate	$9.9\times 10^{-4}$ (${\text{h}}^{\text{-1}}$)^b^	Baccam et al. (2006)
c	Virus clearance rate	0.125 (${\text{h}}^{\text{-1}}$)	Baccam et al. (2006)
μ	Per site mutation rate	$2.3\times 10^{-6}$ (s/n/h)^c^	Sanjuán et al. (2010)
α	Frequency of neut. mutations	0.25	Sanjuán, Moya, and Elena (2004)
sH	Harmonic mean of selection	0.2	Sanjuán (2012)
G	Genome size	13588 (bp)	NIAID
λ	Prob. of no del. mutation	$e^{-(1-\alpha )\mu G/s_{\text{H}}}$	see Equation (1)
$T_{0}$	Initial number of target cells	$4\times 10^{8}$ (cells)	Baccam et al. (2006)
$V_{\text{inoc}}$	Inoculum size	10	McCaw et al. (2011) ^d^

^a^The estimate from Baccam et al. (2006) is 0.167; however, this gives a cell generation time of 6 h (1/δ), which is unrealistic for influenza A virus. Baccam et al. (2006) acknowledge this, and the model has an improved fit when a cell generation time of 12 h is used. Sanjuán (2012) calculates a cell generation time of 10 h (δ=0.1) and notes that it is realistic for a range of eukaryotic viruses. Note that cell generation time is not necessarily equivalent to the viral generation time because many viruses release virions by budding prior to cell lysis.

^b^The estimate from Baccam et al. (2006) is $5\times 10^{-4}$; however, it is more realistic to match the $R_{0}^{\text{wh}}$ value to the Baccam et al. (2006) estimate (see Equation 8), where $R_{0}^{\text{wh}}=11.1$.

^c^The reported value is $2.3\times 10^{-5}$ substitutions per nucleotide site per cell generation (s/n/c) and we convert it to an hourly rate (s/n/h) using the cell generation time 1/δ=10 h.

^d^Reports the number of virions per transmission event inferred from ferret infection data as 4.3±9.8 virions.

#### 2.2.2 Transmission chain

Our model operates on two scales by (1) tracking within-host dynamics of infection and (2) following a chain of disease transmission events between hosts. We simulate infection across multiple hosts by taking an inoculum, or sample of viruses, from a given host to infect the next host in the chain. We assume that transmission occurs when the virus population reaches its peak viral load (Bell et al. 2006). The time of peak viral load $t_{p}$ is estimated by modeling the growth of the virus population and taking the average time to peak population size for 100 replicates.

The probability that a mutant virus is included in the inoculum $V_{\text{inoc}}$ for the next host is equal to the proportion $p_{\text{m}}$ of mutants in the population at the time of transmission:

$$p_{\text{m}}=\frac{W(t_{p})}{V(t_{p})+W(t_{p})}$$

where $t_{p}$ is the time at which the total virus population reaches its peak size. The number of mutant viruses that will make up the inoculum sample is then

$$W(0)∼\text{Binom}(V_{\text{inoc}},p_{m}).$$

The transmission chain stops when a fixation event occurs (i.e., when $W(0)=V_{\text{inoc}}$) or when the maximum number of hosts H is reached.

#### 2.2.3 Calculating the neutral evolutionary rate

We calculate the rate of neutral evolution for the computational model by first estimating the probability of substitution per transmission as follows. The number of hosts in a transmission chain until a fixation event occurs is modeled with a geometric distribution, allowing us to obtain a maximum likelihood estimate of the substitution probability. The likelihood accounts for right-censored data because a transmission chain can reach H hosts without undergoing fixation. The maximum likelihood estimator of the substitution probability per transmission is

$$\hat{p}=\frac{r}{\sum_{i}x_{i}+sH}$$

and the variance of the estimator is the reciprocal of the Fisher information given by

$$I(\hat{p})=\frac{r}{p^{2}}+\frac{\sum_{i}x_{i}-r+sH}{{\left(1-p\right)}^{2}}$$

where r is the number of cases in which a fixation event occurred, s is the number of cases in which no fixation events occurred, $x_{i}$ is the number of hosts in the transmission chain before fixation occurred, and H=2,000 is the maximum number of hosts simulated.

We then convert the substitution probability per transmission to a substitution rate $K_{\text{comp}}$ per year (s/n/y):

(7) $$K_{\text{comp}}=\frac{\hat{p}\times 24\;(\text{h/day})\times 365\;(\text{day/year})}{t_{p}\;(\text{h})}.$$

This value of $K_{\text{comp}}$ can be directly compared with the value calculated by our within-host analytical model, $K_{\text{wh}}$ (Equation 3), or by the deleterious mutation model implemented by Sanjuán (2012), $K_{\text{del}}$ (Equation 4).

## 3 Results

### 3.1 Comparison of model fits for viral evolutionary rates

**Figure 2.**
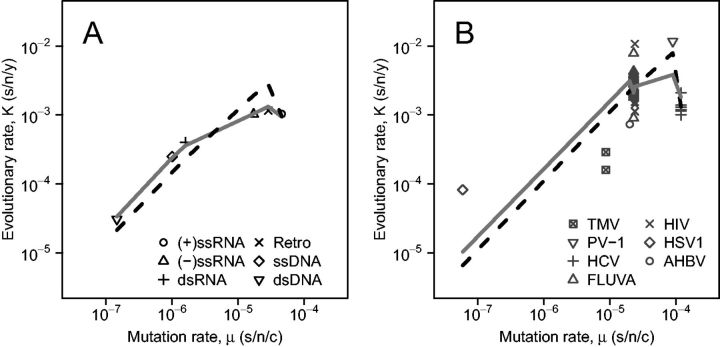
Fitting the within-host analytical and deleterious mutation models to virus data. (A) Log-scale mean evolutionary rates against mutation rates for each Baltimore class (data from Sanjuán (2012) and Sanjuán et al. (2010), respectively). (B) Evolutionary rates against mutation rates for individual viruses. For both (A) and (B), the solid line represents the deleterious mutation model prediction, while the dashed line indicates the prediction from our within-host analytical model. TMV, tobacco mosaic virus ((+)ssRNA); PV-1, poliovirus-1 ((+)ssRNA); HCV, hepatitis C virus ((+)ssRNA); FLUVA, influenza A virus ((-)ssRNA); HIV, human immunodeficiency virus (retro); HSV1, herpes simplex virus 1 (dsDNA); AHBV, avian hepatitis B virus (retro).

We compare our within-host analytical model with the deleterious mutation model implemented by Sanjuán (2012) by fitting our model to the same data analyzed in that study. For these purposes, we reparameterize both models as follows. The deleterious mutation model can be written as $K_{\text{del}}=a\mu e^{-b\mu G}$ where a=gα and $b=(1-\alpha )/s_{H}$. Using the same parameters, our simple within-host model can be written as $K_{\text{wh}}=a\mu (1-1/(\rho e^{-b\mu G}))$. We begin by taking the approach of Sanjuán (2012) by grouping viruses into Baltimore classes. Note that the evolutionary rate values are taken from Sanjuán (2012), while the mutation rates for each class are from Sanjuán et al. (2010). We fit our within-host analytical model in the same manner as in Sanjuán (2012) to obtain $\log_{10}(a)=2.198$, b=3.746, and ρ=13.80. Computing the corrected Akaike Information Criterion (AICc) values, the deleterious mutation model has greater support than our within-host model, with AICc values of −4.16 and 48.04, respectively (Fig. 2A).

While the mutation rate for Baltimore classes might be appropriately grouped (i.e., RNA viruses have much higher mutation rates than DNA viruses), this assumption does not generally apply to within-host reproductive numbers ($R_{0}^{\text{wh}}$). Different viruses within Baltimore classes may have substantially different $R_{0}^{\text{wh}}$ values (see Section 4). Therefore, a more appropriate method is to use estimates of K and μ for individual virus taxa (see Fig. 2B for viruses used). Using this approach, we find $\log_{10}(a)=2.250$ and b=2.132 for the deleterious mutation model, while our within-host model yields $\log_{10}(a)=2.061$, b=2.800, and ρ=28.097. While we find that our model does slightly better than the deleterious mutation model estimate with AICc values of 28.52 and 36.75, respectively (Fig. 2B), the statistical evidence is not strong. The standard error for our parameter estimates is too high to draw firm conclusions, and more data would be needed to resolve this comparison.

### 3.2 Comparison of model predictions for influenza A virus

The within-host dynamics of influenza A virus have been carefully studied, and the parameters underlying these dynamics have been estimated from patient data (Baccam et al. 2006). This allows a more specific comparison of the models for this particular virus without the need for model fitting, in contrast to the analysis of Fig. 2 where we fitted our models assuming a common $R_{0}^{\text{wh}}$ value across a range of viruses. Using a target cell-limited model, Baccam et al. (2006) found the $R_{0}^{\text{wh}}$ for influenza A virus to have an average value of 11.1. We therefore used parameter values in our simulations such that $R_{0}^{\text{wh}}=11.1$ where $R_{0}^{\text{wh}}$ is defined as

(8) $$R_{0}^{\text{wh}}=\frac{p\beta \lambda T_{0}}{c\delta }.$$

Note that λ is a function of the per-site per-generation mutation rate μ (Equation 1) and so we convert the per-hour mutation rate (Table 1) to a per-generation rate via the cell-generation time 1/δ.

**Figure 3.**
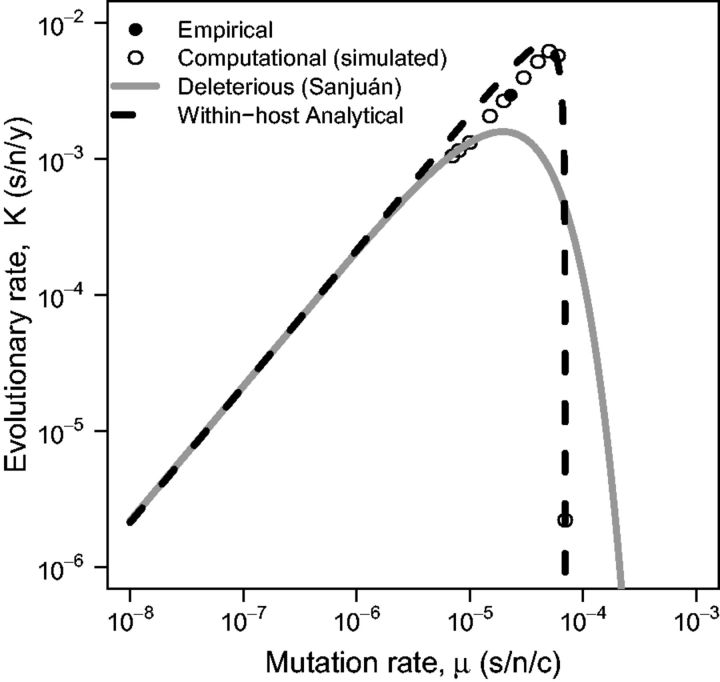
Between-host evolutionary rate K (s/n/y) against the mutation rate μ (s/n/c) for influenza A virus ($R_{0}^{\text{wh}}=11.1$). Data simulated using the computation model (open points, $K_{\text{comp}}$) predicted by the deleterious mutation model implemented by Sanjuán (2012) (solid line, $K_{\text{del}}$), and predicted by our within-host analytical model (dashed line, $K_{\text{wh}}$). Lines represent values calculated from the models based on independently estimated parameters and are not fit to the simulation (open) points. The simulation data closely approximates the reported value of influenza A virus evolutionary rate (closed point). Parameter values for both the computational and analytical models are defined in Table 1.

We simulate the evolution of influenza A virus using empirical parameters (Table 1) across a range of μ values. For comparison, we use the same parameter values for the deleterious mutation model implemented by Sanjuán (2012) (Equation 4) and our within-host analytical model (Equation 3). Figure 3 shows that all models capture the property that the evolutionary rate increases with an increasing mutation rate up to a point, beyond which the evolutionary rate decreases as the population is overcome with deleterious mutations. This trend is consistent with both empirical work and theoretical expectations (Orr 2000; Crotty, Cameron, and Andino 2001; Anderson, Daifuku, and Loeb 2004; Bull, Sanjuan, and Wilke 2007; Sanjuán 2012). The location of the empirical data point for influenza among the simulated points offers strong support for the dynamics of our computational model. The output of the computational model is based on externally estimated parameters and is not fitted to the data point shown in Fig. 3.

Neither the deleterious mutation model (Equation 4) nor the within-host analytical model (Equation 3) match the empirical point perfectly. The deleterious mutation model underestimates the simulated and empirical evolutionary rates, suggesting that mutation rate alone may not be enough to generate an accurate prediction. Our analytical model overestimates the evolutionary rates suggesting that although it does not quite capture reality, within-host dynamics are likely to play a role in the evolutionary rate of viruses. Note that this overestimation is eliminated when a simplified version of our computational model is implemented, that is, when the proportion of mutants for a single infection is used to calculate the substitution rate instead of the probability of fixation across a simulated transmission chain (Supplementary Figure 1). A key observation is that the analytical model and simulations yield a sharper peak in the evolutionary rate than the deleterious mutation model. This can be attributed to the inclusion of within-host dynamics; when $R_{0}^{\text{wh}}$ is high, it can compensate to some degree for an increasing mutation rate.

### 3.3 The effect of within-host dynamics

In the comparison between models in Fig. 3, only the mutation rate is varied; however, the mutation rate is an intrinsic component of $R_{0}^{\text{wh}}$ (Equation 8). Higher values of μ lead to lower $R_{0}^{\text{wh}}$ due to the increased burden of deleterious mutations.

**Figure 4.**
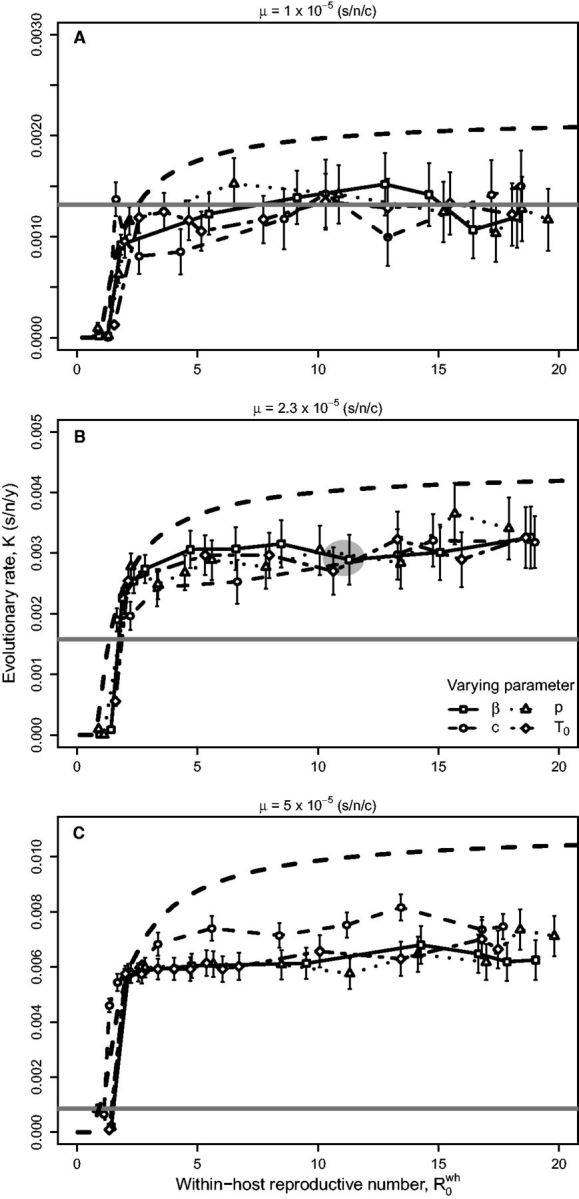
Between-host evolutionary rate $K_{\text{comp}}$ (s/n/y) against within-host reproductive number $R_{0}^{\text{wh}}$. Different specific within-host parameters were varied in the computational model to result in the range of $R_{0}^{\text{wh}}$ values, with mutation rate held constant at (A) $1\times 10^{-5}$, (B) $2.3\times 10^{-5}$, or (C) $5\times 10^{-5}$ (s/n/c). Solid (gray) lines indicate the predicted evolutionary rate by the deleterious mutation model used by Sanjuán (2012), while the dashed (black) lines indicate the predicted rates by our analytical model. Lines represent values calculated from the models using independent parameter estimates (Table 1) and are not a fit to the simulation points. The gray shaded area in (B) represents the empirical value of influenza (one standard error in each direction). Within-host parameter variables are cell infection rate β, virus production rate p, virus clearance rate c, and initial number of target cells $T_{0}$.

To understand the effect of other viral within-host parameters on the evolutionary rate $K_{\text{comp}}$, we held the mutation rate constant and varied $R_{0}^{\text{wh}}$ in our computational model by changing the individual parameters: virus infection rate β, virus production rate p, virus clearance rate c, and initial target cell population $T_{0}$ (Equation 8). By varying one parameter at a time, we see that the evolutionary rate increases with increasing $R_{0}^{\text{wh}}$ and approaches a plateau (Fig. 4). The evolutionary rate $K_{\text{comp}}$ is most sensitive to $R_{0}^{\text{wh}}$ when $R_{0}^{\text{wh}}$ is low; here, a slight change in $R_{0}^{\text{wh}}$ can have a larger impact on the probability that a mutant survives stochastic loss ($1-1/R_{0}^{\text{wh}}$). We find that varying a specific parameter does not change the relationship, suggesting that $R_{0}^{\text{wh}}$ is an effective predictor of $K_{\text{comp}}$—that is, no particular combination of parameters (i.e., β, p, c, and $T_{0}$) drives the result. We also used three different mutation rates ($1\times 10^{-5}$, $2.3\times 10^{-5}$, and $5\times 10^{-5}$ (s/n/c)) to demonstrate that large $R_{0}^{\text{wh}}$ values can facilitate high rates of evolution at high mutation rates.

The finding that evolutionary rate can increase with an increasing $R_{0}^{\text{wh}}$ indicates that the mutation rate may not be sufficient in explaining $K_{\text{comp}}$. The prediction of the deleterious mutation model yields a constant K value in Fig. 4 (solid line) because it does not explicitly account for $R_{0}^{\text{wh}}$. Note that the higher estimates of K in the analytical model are consistent with other parameter sets (see Fig. 3, with evolutionary rate plotted on a log scale compared with Fig. 4 plotted on a linear scale.).

### 3.4 The effect of between-host dynamics

**Figure 5.**
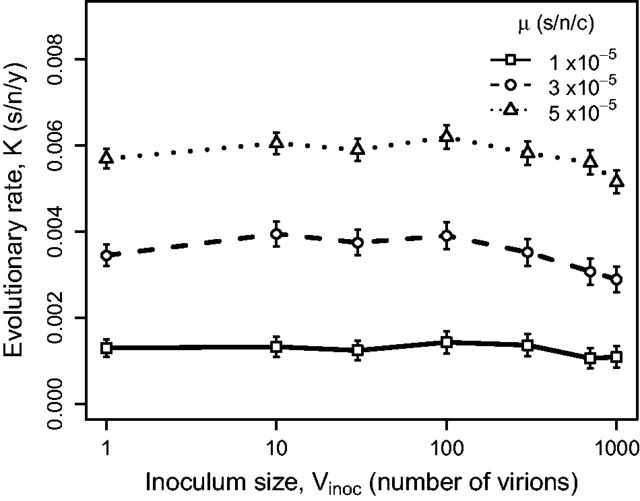
Between-host evolutionary rate $K_{\text{comp}}$ (s/n/y) as a function of inoculum size $V_{\text{inoc}}$ for various values of μ. Other parameter values are given in Table 1. The mean evolutionary rate does not change with different $V_{\text{inoc}}$.

The computational model allows us to investigate the effect of inoculum size on the rate of molecular evolution. Under neutral theory, the rate of molecular evolution is a function of the mutation rate but not the population size; thus the inoculum size is not expected to influence K. We used different inoculum sizes to simulate the evolution of influenza A virus along a transmission chain. We find that the mean evolutionary rate is not affected by inoculum size ($V_{\text{inoc}}$), which is consistent with standard neutral theory (Fig. 5). Note that under the nearly neutral theory, evolutionary rate is influenced by the effective population size because it determines the proportion of effectively neutral mutations. However, we do not alter the underlying mutation rate, thus preserving the expectation of an evolutionary rate independent of population size.

## 4 Discussion

The high mutation rates of viruses provide an opportunity to study evolutionary processes on short timescales and to examine the factors that cause variation in evolutionary rates. Sanjuán (2012) showed that while the neutral theory can explain the evolutionary rates seen for slowly mutating viruses, the linear relationship between mutation rate and substitution rate breaks down as mutation rates increase. In populations of viruses with high mutation rates—in particular, RNA viruses—transient deleterious mutations are common and slow down the fixation of neutral or advantageous mutations (Sanjuán 2012). Population genetics theory suggests that in addition to the mutation rate and the presence of deleterious mutations, population dynamics can also influence neutral substitution rates (Waxman 2012). In line with this idea, we have shown here that within-host dynamics are a key determinant of the rate of viral evolution. We quantified this by developing a model that incorporates both deleterious mutations and within-host dynamics parameterized explicitly through the within-host basic reproductive number $R_{0}^{\text{wh}}$ (Equation 3).

Reanalysis of Sanjuán’s (2012) data provides some support for our model, but more data are required for a definitive analysis. At this stage, accurate empirical measurements are unfortunately difficult to come by. Methods of mutation rate estimation are variable and complex (Sanjuán et al. 2010), while rates of molecular evolution are difficult to estimate precisely because they are variable (Jenkins et al. 2002). Additionally, empirical measurements of $R_{0}^{\text{wh}}$ values are scarce due to current methods for obtaining within-host parameter values requiring patient data in a controlled setting (Perelson et al. 2005; Baccam et al. 2006; Perelson and Ribeiro 2008). Despite the paucity of empirical estimates, we expect viruses to have different $R_{0}^{\text{wh}}$ values based on variation in life cycle properties such as the replication rate, the type and number of target cells available, the efficiency of cell entry, the rate at which the immune system recognizes and clears infection, how transmission occurs, and the number of virions required to establish an infection.

In comparison to the deleterious mutation model used by Sanjuán (2012), our model incorporating viral growth can maintain the linear relationship between mutation rate and evolutionary rate for longer as the mutation rate rises. This is because the viral growth rate can compensate for a higher deleterious load and thus modulate the height of the peak. This flexibility in our model is important because it allows us to make different predictions for individual viruses, particularly about their potential peak evolutionary rate. We note that in Sanjuán’s (2012) analysis, the observed rates of evolution for three out of four viruses (hepatitis C virus, poliovirus 1, and influenza A virus) are higher than the predicted curve under the deleterious mutation model (Fig. 3 in Sanjuán (2012)). This result is also consistent with theoretical models of deleterious mutations in growing populations (Waxman 2011; Gazave et al. 2013). We note that model fitting to Sanjuán’s data involved assuming common $R_{0}^{\text{wh}}$ values and similar levels of deleterious mutations, which may in fact vary considerably between viruses. However, in the case of influenza A virus for which parameter estimates are available (Baccam et al. 2006), we find that our computational model matches the empirical value more closely, suggesting that including within-host dynamics is a step in the right direction.

Our results describing the effect of the within-host reproductive number have several implications for virus evolution. First, the effect of $R_{0}^{\text{wh}}$ depends on the mutation rate, so that increased $R_{0}^{\text{wh}}$ will not necessarily result in a substantial increase in evolutionary rate. It is only at high mutation rates that an increase in $R_{0}^{\text{wh}}$ will increase the evolutionary rate (Fig. 4). Second, viruses that evolve a greater $R_{0}^{\text{wh}}$ can tolerate higher mutation rates; an increase in $R_{0}^{\text{wh}}$ may be a prerequisite for evolving higher mutation rates. While we do not consider the evolution of mutation rates in this study, the virus growth rate and the mutation rate are recognized to be intimately connected (Furió, Moya, and Sanjuán 2007; Belshaw et al. 2008; Regoes, Hamblin, and Tanaka 2013). It would be interesting to experimentally test whether there is strong selective pressure to maintain $R_{0}^{\text{wh}}$ once viruses reach the maximum mutation rate value before passing the extinction threshold. Third, our results suggest a possible source of variation in rates of viral molecular evolution, namely variation in the within-host reproductive value. Such variation may contribute to the departure from a strict molecular clock observed among numerous viruses (Jenkins et al. 2002).

Within-host factors other than $R_{0}^{\text{wh}}$ may influence the rate of virus evolution. The importance of understanding the role of within-host replication is highlighted by a recent study by Hicks and Duffy (2014) which found that viruses infecting more rapidly proliferating epithelial cells have higher rates of evolution. Interpreting cell tropism in the context of $R_{0}^{\text{wh}}$ lends support to the importance of within-host dynamics because different target cells have different turnover rates (Savage et al. 2007) and may have varying cell infection and death rates, and impose varying rates of virus production and clearance.

Between-host factors can also influence evolutionary rates (Berry et al. 2007; Frost and Volz 2010; Scholle et al. 2013). For example, human T-cell lymphotropic virus type II has different rates of evolution depending on whether it was spread by needle sharing or by breast-feeding (Salemi et al. 1999). Additionally, different rates of evolution were found for flaviviruses, depending on whether they were tick- or mosquito-borne (Zanotto et al. 1996). Other studies have found that HIV-1 evolutionary rates are influenced by whether transmission occurs by intravenous drug use or spread by heterosexual individuals, with the former resulting in lower rates and mixed epidemics resulting in intermediate rates (Berry et al. 2007). Additionally, the within-host evolutionary processes differing between heterosexual and male–male partners were found to influence between-host evolutionary rates, with the latter group resulting in higher rates (Vrancken et al. 2015). Here, we have examined one aspect of transmission, namely the inoculum size. Inoculum size plays a relevant role in the spread of viruses, with small inoculum sizes representing transmission of aerosolized viruses (e.g. sneezing), while large inoculum sizes represent spread via direct contact (e.g. mucus). Note, however, that this is not always the case: HIV is spread by direct contact but most infections are established by a single infectious unit or a small number of variants (Abrahams et al. 2009). The population bottleneck imposed by the inoculum may influence traits under selection such as virulence (Bergstrom, McElhany, and Real 1999). Here, however, it has no influence over the rate of neutral evolution.

For future work, it may be useful to include different transmission modes, which have been implicated in affecting rates of evolution. In a comprehensive study of how transmission mode influences the rate of synonymous substitutions in RNA viruses, it was found that viruses that spread rapidly among hosts (i.e., aerosol, contagious, and fecal-oral routes) had higher substitution rates than viruses that spread by blood, bite, or vector routes (Hanada, Suzuki, and Gojobori 2004). In fact, arboviruses have been found to have significantly lower substitution rates than directly transmitted viruses (Hanada, Suzuki, and Gojobori 2004; Combe and Sanjuán 2014; Hicks and Duffy 2014). Combe and Sanjuán (2014) suggest that this difference may be explained by a lower viral mutation rate in insect cells compared with mammalian cells, but it may be interesting to examine the contribution of additional factors.

To better understand the rate of evolution in chronic viral infections, our model would need to be modified appropriately because it specifically models acute infections. For example, in an extended model, early clearance would not occur and between-host transmission would occur any time rather than at the peak of infection. In this case, we expect $R_{0}^{\text{wh}}$ not to play a strong role in the rate of evolution. The relationship between within-host and between-host evolution has previously been modeled to understand virus evolution, particularly for such chronic infections (see Pybus and Rambaut 2009). Chronic viruses may have very different rates of evolution at the within-host and between-host scales (Gray et al. 2011; Lythgoe and Fraser 2012). Traits favored at the within-host level may result in reduced fitness at the between-host level. For example, in HCV, an increased viral replication rate enhances fitness at the within-host level but slowly replicating viruses lead to more secondary infections and have a higher epidemiological fitness (Luciani and Alizon 2009). Similarly, as within-host competition increases in HIV infections, the epidemiological fitness decreases (Lythgoe, Pellis, and Fraser 2013).

Our computational model can be extended in a number of other ways. It would be interesting to consider the effect of selection in more depth by including adaptive mutations (i.e., positive selection). More complex immune responses to infection within a single host could be considered with different levels of immunity for different hosts in a transmission chain. Our modeling framework can be used to explore other viruses and compare the outcomes to influenza A virus. Overall, viral infection dynamics are highly complex and there is considerable scope for future work. The models presented in this article provide a basis for understanding how some aspects of the viral life cycle affect evolutionary dynamics.

## Data availability

All data used for model fitting can be found in Sanjuán et al. (2010) and Sanjuán (2012). C code and output for the computational model are available upon request.

## Supplementary data

Supplementary data is available at *VEVOLU Journal* online.

Supplementary Data
