# Supplementary material for: Connecting within-host dynamics to the rate of viral molecular evolution
Source: Virus Evol. 2015 Oct 2;1(1):vev013. doi: 10.1093/ve/vev013 (PMC5014490; doi:10.1093/ve/vev013)
Supplement: Supplementary Data [file Supplementary_Figure_1.pdf]

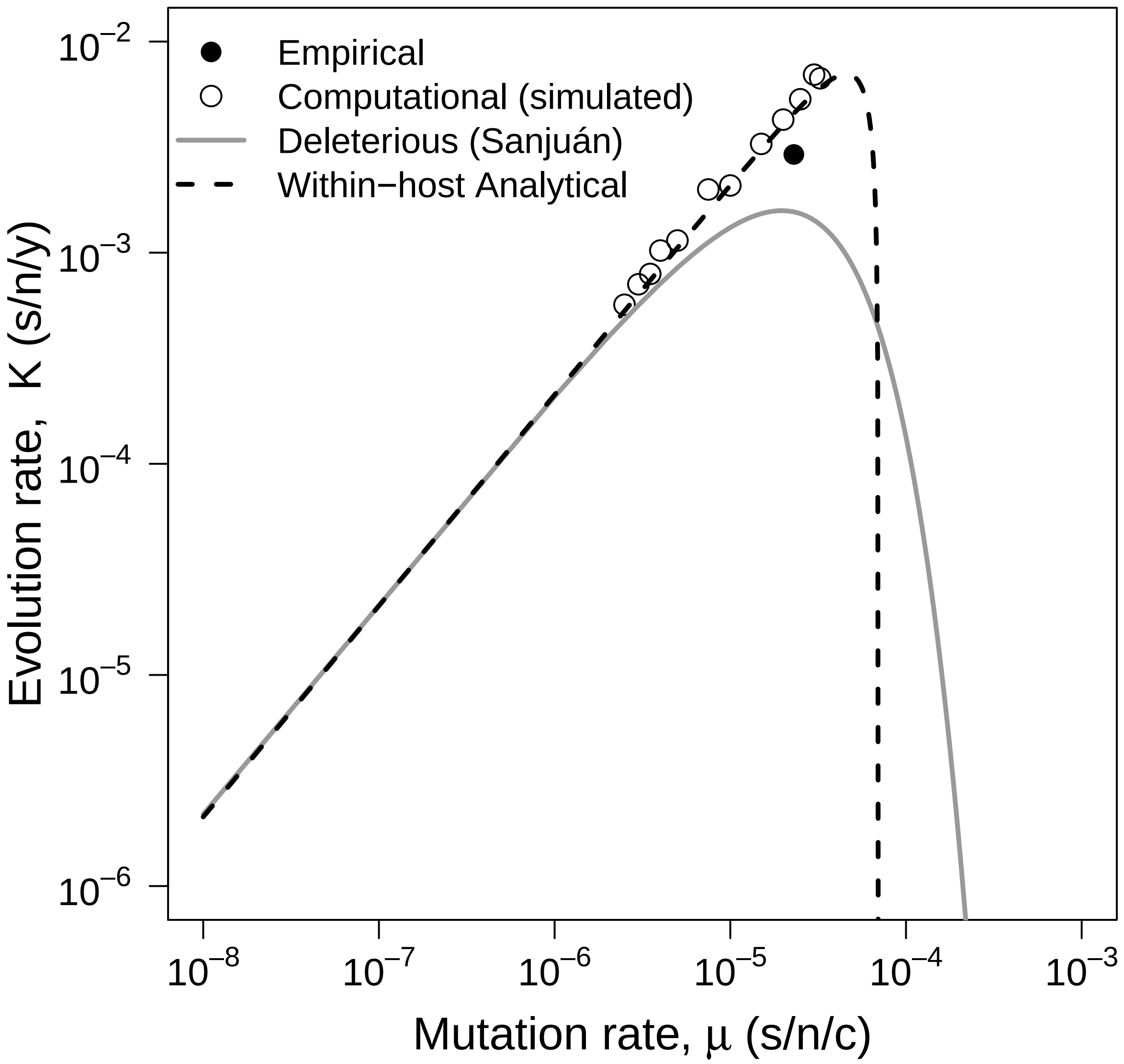

Empirical
Computational (simulated)
Deleterious (Sanjuán)
Within−host Analytical
Evolution rate, K (s/n/y)
Mutation rate, μ (s/n/c)
10−2
10−3
10−4
10−5
10−6
10−8
10−7
10−6
10−5
10−4
10−3

Figure S1: Between host evolution rate K (s/n/y) against the mutation rate (s/n/c) for influenza A virus (R_0^{wh} = 11:1). Open points were computed using a simplified version of the computation model that approximates the analytical model: the evolution rate K_{comp} is calculated using the proportion of mutants at the peak of infection (Equation 7) instead of simulating a transmission chain (as in Figure 3). Lines indicate predictions of the deleterious mutation model implemented by Sanjuan (2012) (solid line, K_{del}), and of our within-host analytical model (dashed line, K_{wh}). Lines represent values calculated from the models based on independently estimated parameters and are not fitted to the simulation (open) points. Parameter values for both the computational and analytical models are defined in Table 1.
